# Molecular Network Analysis of HBV Persistent Infection from the Perspective of Whole Transcriptome

**DOI:** 10.3390/biom15121678

**Published:** 2025-12-01

**Authors:** Qiuping Chen, Congying Tang, Haiyang Hu, Yichen Peng, Jibin Liu, Peijie Wu, Quansheng Feng, Yuming Jiang, Baixue Li

**Affiliations:** 1 School of Basic Medical Sciences, Chengdu University of Traditional Chinese Medicine, Chengdu 611137, China; qiuqiuchen0921@126.com (Q.C.);; 2 School of Clinical Medicine, Chengdu University of Traditional Chinese Medicine, Chengdu 611137, China; 3 Key Laboratory of Digital Protection for Unearthed Medical Literature and Cultural Relics, Chengdu University of Traditional Chinese Medicine, Chengdu 611137, China; 4 School of Computer Science, Sichuan University (School of Software, School of Intelligent Science and Technology), Chengdu 610207, China

**Keywords:** hepatitis B virus, recombinant rcccDNA, chronic hepatitis B, whole transcriptomics

## Abstract

(1) Background: After HBV infection, viral transcripts and host RNA form a multi-layered interwoven regulatory network. However, a comprehensive map encompassing mRNA, miRNA, lncRNA, and circRNA is still lacking. This absence complicates the systematic explanation of the molecular mechanisms driving immune escape and metabolic reprogramming during the persistent infection stage. (2) Methods: In this study, we established a mouse model of chronic HBV infection and analyzed the differential expression of mRNA, miRNA, lncRNA, and circRNA through whole transcriptome sequencing (WTS). We constructed a competing endogenous RNA (ceRNA) network to systematically evaluate the overall impact of HBV on the host’s immune-metabolic pathways. (3) Results: RNA sequencing results indicated that HBV infection significantly up-regulated 194 mRNAs, 18 miRNAs, 184 lncRNAs, and 28 circRNAs, while down-regulating 42, 16, 122, and 31 corresponding transcripts, respectively. The differentially expressed genes were primarily enriched in pathways related to metabolism, immunity/inflammation, and signal transduction-ligand receptor interactions. Furthermore, the competitive endogenous RNA networks of lncRNA-miRNA-mRNA and circRNA-miRNA-mRNA constructed on this basis further identified miR-185-3p as a key core node. (4) Conclusions: In this study, based on whole transcriptome data, the gene expression profiles of rcccDNA/Ad-infected Alb-Cre transgenic mice (chronic HBV infection model) and normal Alb-Cre mice were systematically compared, and the core regulatory factor miR-185-3p of key differentially expressed genes was screened. The microRNA is expected to provide a new target for the precise treatment of chronic hepatitis B by targeted intervention of viral replication and high liver inflammation.

## 1. Introduction

Chronic hepatitis B virus (HBV) infection remains a significant global public health issue. In 2022, approximately 254 million individuals worldwide were estimated to be chronically infected with HBV [1], leading to around 1 million deaths annually due to decompensated cirrhosis, liver failure, or hepatocellular carcinoma resulting from the progression of HBV infection [2]. Current treatment primarily involves the use of nucleoside (acid) analogs (NAs) and interferon-α (IFN-α) [3,4]. Although these treatments can effectively inhibit viral replication, complete eradication of the virus remains challenging. The primary barrier to achieving this goal is the persistence of covalently closed circular DNA (cccDNA) within the nuclei of liver cells [5]. On one hand, cccDNA has a long half-life and can maintain low-level replication even after prolonged antiviral treatment; on the other hand, the stable viral reservoir serves as a transcription template and is resistant to existing drugs, thereby sustaining chronic infection and promoting cirrhosis and liver cancer. Furthermore, HBV can enhance its persistence through immune evasion mechanisms. To date, the dynamic changes in the local immune microenvironment of the liver during chronic HBV infection remain unexplored. This gap in knowledge primarily arises from the fact that most existing studies depend on peripheral blood samples from HBV-infected patients, whereas local liver tissue data are still scarce. The liver exhibits a unique immunosuppressive microenvironment and alterations in metabolic regulation, which peripheral blood cannot accurately and comprehensively reflect regarding the specific immune regulation and metabolic status of the host liver. This limitation indeed hinders researchers’ ability to gain a deeper understanding of the mechanisms underlying intrahepatic immune regulation and metabolic changes during HBV persistent infection.

HBV is a hepatotropic DNA virus that exclusively infects human hepatocytes and does not infect murine models. Consequently, the absence of an ideal animal model for persistent HBV infection, which can continuously express HBV and maintain a human-like immune environment over extended periods, has hindered the investigation of the mechanisms underlying viral persistence through animal studies. In earlier research, a Cre/loxP-mediated site-specific recombination strategy was employed to induce the formation of cccDNA from pre-formed particles, leading to the establishment of an extended HBV viremia model in mice via high-pressure tail vein injection [6]. However, mice naturally lack susceptibility to HBV, rendering them incapable of simulating the true infection process and immune response observed in patients. Consequently, this model is challenging to utilize for studying the key mechanisms of viral persistent replication, immune regulation, and metabolic changes. This limitation hinders the preclinical verification and optimization of treatment strategies related to HBV persistent infection. Deng et al. were the first to establish a chronic hepatitis model based on the persistent infection of HBV recombinant covalently closed circular DNA (rcccDNA). They achieved this by inducing cccDNA epigenetic silencing and utilizing an adenovirus vector for the delivery of rcccDNA in the mouse liver. This study addresses the limitation of achieving chronic HBV infection in mice with normal immune function and provides a novel model for further understanding the mechanisms of viral persistence and optimizing treatment strategies.

**Figure 1:**
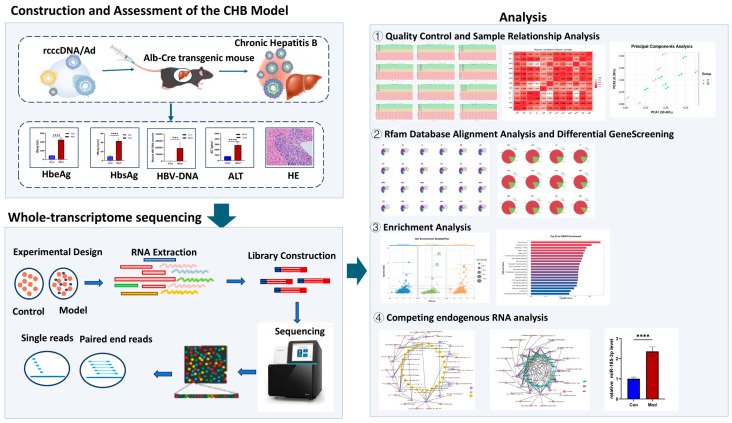
Schematic overview of the study design.

Despite the extensive focus of numerous omics studies on the peripheral immune response in individuals infected with HBV, a comprehensive transcriptome analysis of liver tissue in relevant persistent infection animal models remains incomplete. This lack of analysis hampers the ability to differentiate between peripheral and intrahepatic specific immune-metabolic changes. This study utilized the HBV-rcccDNA persistent infection mouse model established by Deng’s team (immunocompetent) [7]. For the first time, we conducted comprehensive transcriptome sequencing of the liver tissue from this model (Figure 1), mapping the host gene expression profile against the backdrop of continuous viral replication. This research provides foundational data for the subsequent identification of intrahepatic restriction biomarkers, the elucidation of the molecular mechanisms underlying HBV chronic infection, and the exploration of potential therapeutic targets.

## 2. Materials and Methods

### 2.1. Animals

Thirty specific-pathogen-free (SPF) male Alb-Cre mice (18–20 g) were obtained from Guangzhou Ruige Biotechnology Co., Ltd. (Guangzhou, China). License numbers: SYXK (Guangdong) 2023-0343 (experimental animal use) and SCXK (Guangdong) 2021-0059 (animal production). Each cage housed five animals, with ad libitum access to food and water. The light cycle was maintained at 12 h of light and 12 h of darkness. Air exchange was conducted at a rate of at least 10 times per hour. The room temperature was kept between 20 and 24 °C, with a daily fluctuation not exceeding 1 °C, and relative humidity was maintained between 30% and 70%. After seven days of adaptive feeding, the animals entered the experiment without any abnormalities. All animal experiments were approved by the Animal Welfare Ethics Committee of our institution (approval code: 2022–31). Fifteen mice were randomly selected as the control group (group C), while the remaining 15 mice were used to construct a rcccDNA model of HBV persistent infection according to the method of Deng et al. [7]; namely, the model group (group M). The specific procedure is as follows: recombinant adenovirus Ad/rcccDNA carrying ayw subtype (*genotype D*) HBV genome was injected into *Alb-Cre* transgenic mice via tail vein.

### 2.2. Quantification of Peripheral Blood ALT, HBsAg, and HBeAg Using ELISA

According to the manufacturer’s instructions, the corresponding indices in peripheral blood serum were quantified using the mouse ALT ELISA kit (Shanghai Yuanju Biotechnology Center, Shanghai, China), HBsAg ELISA kit (Shanghai Yuanju Biotechnology Center), and mouse HBeAg ELISA kit (Shanghai Yuanju Biotechnology Center). The absorbance was measured at 450 nm using a SpectraMax Molecular Devices spectrophotometer (Molecular Devices, San Jose, CA, USA), with background correction performed at 540 nm and 570 nm.

### 2.3. RT-qPCR-Based Quantification of HBV DNA in Liver Tissue

The mortar, precooled with liquid nitrogen, was placed on dry ice. Subsequently, 20–30 mg of liver tissue was quickly ground into a fine powder and transferred in its entirety to a 1.5 mL centrifuge tube. Following the instructions provided with the tissue genomic DNA extraction kit (Beijing Yuntai Biotechnology Co., Ltd., Beijing, China), the processes of cleavage, column passing, and elution were performed, ultimately yielding genomic DNA eluted in 50 µL of nuclease-free water. A 1 µL DNA template was added to a 20 µL qPCR system for amplification, while the remaining DNA was stored at −80 °C (Table 1).

### 2.4. RT-qPCR-Based Quantification of miR-185-3p in Liver Tissue

Total RNA was extracted using the All-Gold TransZol Up Plus RNA Kit (Beijing TransGen Biotech Co., Ltd., Beijing, China). Approximately 30 mg of liver tissue was homogenized with 1 mL of TransZol Up (Beijing TransGen Biotech Co., Ltd., Beijing, China). Following this, phase separation, genomic DNA removal, and rinsing were performed according to the manufacturer’s instructions, and the RNA was finally eluted with 30–50 μL of RNase-Free Water. The extracted RNA was quantified using a NanoDrop spectrophotometer (Beijing TransGen Biotech Co., Ltd., Beijing, China), yielding an A260/A280 ratio between 1.8 and 2.1. The reverse transcription reaction was conducted using the Novozymes miRNA first-strand cDNA synthesis kit (MR101), incorporating 100 ng of total RNA and specific stem-loop primers for miR-185-3p and U6 in a 20 μL reaction system. The reaction conditions were set at 25 °C for 5 min, 50 °C for 45 min, and 85 °C for 2 min. Subsequently, the diluted cDNA served as a template for quantitative PCR (qPCR) using the All-Gold TransStart Tip Green qPCR SuperMix (Beijing TransGen Biotech Co., Ltd., Beijing, China). The qPCR reaction system was also 20 μL, containing 1 × SuperMix, 0.2 μM miR-185-3p or U6-specific forward primers, and universal reverse primers. The amplification protocol consisted of an initial denaturation at 94 °C for 30 s, followed by 40 cycles of denaturation at 94 °C for 5 s and annealing/extension at 60 °C for 30 s. Each sample was analyzed in triplicate, and amplification specificity was confirmed via melting curve analysis. Finally, the relative expression of miR-185 was calculated using the 2^(−ΔΔCt)^ method, with U6 serving as the internal reference (Table 2).

### 2.5. HE Staining to Evaluate the Pathological Injury of Liver Tissue

Fresh mouse liver tissue samples were fixed in 4% paraformaldehyde for 24 h and dehydrated using a gradient of ethanol. The transparent tissues were embedded in melted wax and subsequently sectioned using a microtome. The sections were dewaxed with xylene three times (for 5 min each), followed by rehydration in 100%, 95%, 85%, and 75% ethanol for 3 min each, and soaked in distilled water for 2 min. After hematoxylin staining for 5 min, the excess dye was removed by washing with water. The differentiation solution was applied for 10 s, followed by two washes with running water (3 min each). The sections were then returned to warm water to achieve a blue coloration for approximately 1 min. Eosin staining was performed for 1 min, after which the slides were rapidly dehydrated using a gradient of 75%, 85%, 95%, and 100% ethanol. The slides were then cleared in xylene twice (for 1 min each). Finally, neutral resin was used to seal the slides. TIFF images of the entire slides were obtained using a high-resolution pathological scanner to evaluate the degree of inflammation.

### 2.6. RNA Extraction and Library Construction

Total RNA was extracted from liver tissue (20 mg) from each group of mice using Trizol reagent (Thermo Fisher Scientific Inc., Waltham, MA, USA), following the manufacturer’s protocol. The Bioanalyzer 2100 and RNA 6000 Nano LabChip Kit (Agilent, Santa Clara, CA, USA, 5067-1511) were utilized to assess the quantity and purity of total RNA, with a required RIN value of greater than 7. Take approximately 5 µg of total RNA and remove ribosomal RNA following the instructions provided in the Ribo-Zero Gold rRNA Removal Kit manual (Illumina, MRZG12324, San Diego, CA, USA). Subsequently, fragment the RNA using the NEBNext^®^ Magnesium RNA Fragmentation Module (NEB, E6150S, Ipswich, MA, USA) under high temperature and divalent cation conditions. Using SuperScript™ II reverse transcriptase (Invitrogen, 1896649, Waltham, MA, USA), fragmented RNA was reverse transcribed to synthesize the first strand of cDNA. Subsequently, the second strand of DNA containing U labels was synthesized using *E. coli* DNA polymerase I (NEB, m0209, USA), RNase H (NEB, m0297, USA), and dUTP solution (Thermo Fisher, R0133, USA). After adding an A tail to the blunt end of the double-stranded DNA, it is subsequently connected to an index adapter that features a T overhang. The ligation products were then screened for fragments ranging from 300 to 600 base pairs using AMPure XP magnetic beads. After treating the uracil-containing second strand with the thermosensitive UDG enzyme (NEB, M0280, USA), PCR amplification was conducted. The average insert length of the final cDNA library was 300 ± 50 bp.

### 2.7. RNA Sequencing and Bioinformatics Analysis

Paired-end sequencing (PE150) of 2 × 150 bp was performed using the Illumina NovaSeq™ 6000 platform (Lianchuan Biotechnology Co., Ltd., Hangzhou, China). Utilizing CASAVA/Basecall_T7_GPU_1.2.0.26_Centos, the raw image data files generated by sequencing were converted into raw sequencing data (RawData) through base calling analysis. Subsequently, fastp (v0.20.0) was employed to filter low-quality sequencing data, and the high-quality sequencing data were aligned to the mouse reference genome using HISAT2 (v2.1.0). Gene expression levels were quantified as transcript fragments per kilobase per million aligned reads (FPKM). All data reached the Q30 quality threshold, indicating that the samples and sequencing data generated in this study were of high quality and suitable for subsequent bioinformatics analysis. The DESeq2 (v1.30.1) software was utilized to identify differentially expressed genes, including *DEmRNA*, *DEmiRNA*, *DELncRNA*, and *DEcircRNA*, based on the criteria of |log2FoldChange| ≥ 1 and a corrected *p*-value < 0.05. Expression profile differences between groups were assessed using the prcomp function for principal component analysis (PCA). Volcano plots and heat maps were generated with the ggplot2 package, while the OmicStudio tools at https://www.omicstudio.cn/tool (accessed on 20 October 2025) was utilized to conduct Gene Ontology (GO) and Kyoto Encyclopedia of Genes and Genomes (KEGG) enrichment analyzes of the differential genes. A variety of algorithms were employed to predict target pairs of *DEmRNA*, *DEmiRNA*, *DELncRNA*, and *DEcircRNA*, and target pairs associated with HBV infection were identified. Two sets of *ceRNA* networks, specifically *lncRNA–miRNA–mRNA* and *circRNA–miRNA–mRNA*, were constructed and visualized using Cytoscape v3.9.1.

### 2.8. Statistical Analysis

The experiments were conducted six times (*n* = 6), and the results are presented as mean ± standard deviation (SD). Comparisons between the two groups were performed using a two-tailed *t*-test with GraphPad Prism 10. A *p*-value of <0.05 indicates that the difference is statistically significant (* *p* < 0.05, ** *p* < 0.01, *** *p* < 0.001, **** *p* < 0.0001).

## 3. Results

### 3.1. Assessment of Viral Replication and Hepatic Inflammation in a Mouse Model of Chronic HBV Infection

**Figure 2:**
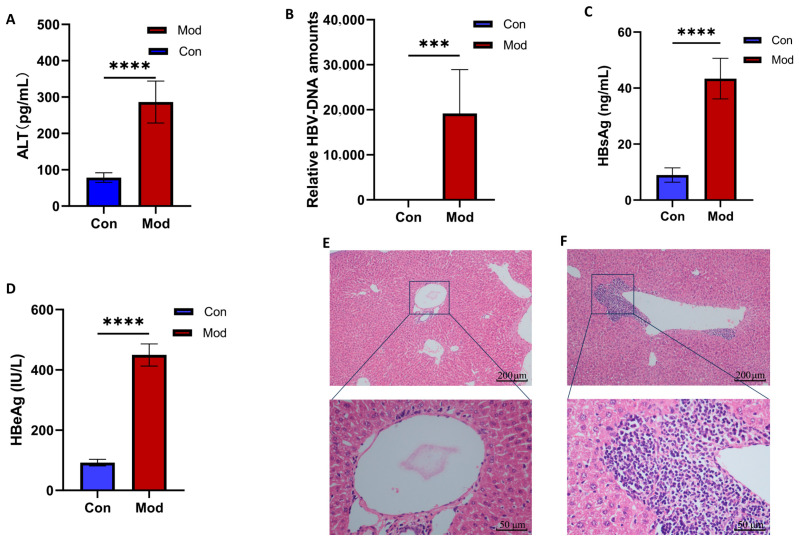
The construction and evaluation of a mouse model for persistent HBV infection. (**A**) ELISA was employed to measure ALT expression levels in the peripheral blood of mice with persistent HBV infection; (**B**) qRT-PCR was utilized to assess the relative expression of HBV-DNA in the livers of these mice (relative HBV DNA level calculated using genomic GAPDH as internal reference); (**C**) ELISA was again used to evaluate HBsAg expression levels in the peripheral blood of mice with persistent HBV infection (the positive judgment threshold was 15.94 ng/m, 8.98 ± 2.32 ng/mL in the control group and 43.38 ± 6.61 ng/m in the model group); (**D**) ELISA was used to detect HBeAg expression levels in the peripheral blood of mice with persistent HBV infection (the positive judgment threshold was 122.32 IU/L, the control group was 92.14 ± 10.06 IU/L, and the model group was 449.66 ± 33.52 IU/L); (**E**) Hematoxylin and eosin (HE) staining was performed to examine liver tissue damage in the control group; (**F**) HE staining was also conducted to observe liver tissue damage in the model group. (*** *p* < 0.001, **** *p* < 0.0001).

To elucidate the biological mechanisms underlying chronic HBV infection in the host, rcccDNA was efficiently delivered into the mouse liver via tail vein injection, successfully constructing an animal model that simulates human chronic HBV infection. Our analysis revealed that ALT levels in the peripheral blood of chronic HBV-infected mice were significantly elevated compared to those in the blank control group (Figure 2A). Additionally, serum levels of HBsAg (Figure 2C) and HBeAg (Figure 2D) increased to 44 ng/mL and 450 IU/mL, respectively. Furthermore, we observed that HBV-DNA expression in liver tissue remained elevated for an extended period following infection (Figure 2B). To further assess the damaging effects of Ad/rcccDNA on the liver, we performed HE staining on liver tissue. The results indicated that, compared to the blank control group (Figure 2E), mice with chronic HBV infection (Figure 2F) exhibited significant infiltration of inflammatory cells around the larger hepatic vein branches, disordered arrangement of adjacent hepatocytes, and mild edema, confirming that the persistent HBV infection mediated by Ad/rcccDNA has induced substantial inflammatory liver damage.

### 3.2. Identification of DEmRNAs

**Figure 3:**
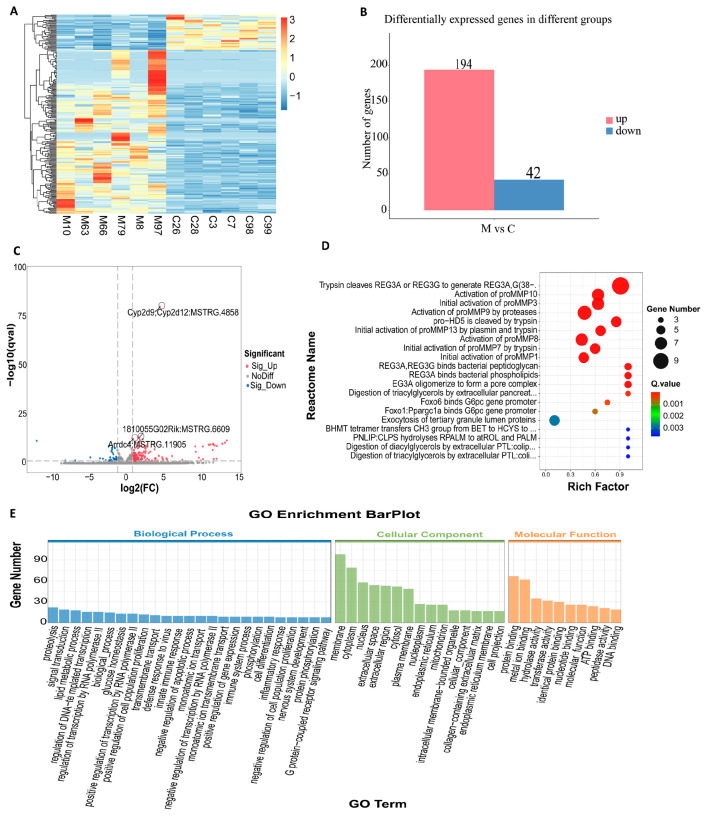
Identification of *DEmRNAs*. (**A**) The clustering heat map of *DEmRNAs* (The color bar scale is the z-score value, which represents the deviation multiple of the relative mean value of the original data after standardization, where 0 is the mean value, positive values 1, 2, and 3 represent 1, 2, and 3 standard deviations higher than the mean value, respectively, and −1 represents 1 standard deviation lower than the mean value); (**B**) The overall statistical histogram of *DEmRNAs*; (**C**) The volcano plot depicting differential gene expression in the comparison group; (**D**) Reactome Enrichment ScatterPlot; (**E**) The GO enrichment histogram.

After the removal of adapters and low-quality sequences, the proportion of clean reads exceeded 90%, with base accuracy for Q20 and Q30 surpassing 98%. This indicates that both the quantity and quality of the sequencing data are adequate for subsequent analyzes (Table S1). The results of the differential analysis are presented as follows: the heat map of differentially expressed *mRNA (DEmRNA)* indicates that the samples in the comparison group clustered into two main branches, suggesting that the differentially expressed *mRNA* possesses a robust ability to distinguish between the groups (Figure 3A). In comparison to group C, a total of 238 *DEmRNAs* were identified in group M, of which 194 genes were significantly up-regulated and 42 genes were significantly down-regulated (Figure 3B). It is worth noting that a new transcript (StringTie ID: *MSTRG.4858*) located in the intergenic region between *Cyb29* and *Cyp2d12* was significantly up-regulated (Figure 3C), suggesting that it may play a key role in viral clearance and hepatocyte stress response. To explore the biological functions of differentially expressed *mRNAs*, we first identified the most significant representative entries using GO BarPlot Level (Figure 3E): proteolysis (GO:0006508), membrane structure (GO:0016020), and protein binding activity (GO:0005515). GO _ ZScore _ BubblePlot. Q (Figure S1A) focused on three up-regulated key nodes: disruption of cell wall in another organism (GO: 0052006), extracellular space (GO: 0005615) and serine-type endopeptidase activity (GO: 0004252). We further expanded the analysis to the top 20 most significant Gene Ontology (GO) terms (Figure S1C) and found that ‘extracellular space’ (GO: 0005615) and ‘extracellular region’ (GO: 0005576) ranked at the top. This observation suggests that differentially expressed genes are predominantly concentrated in the extracellular microenvironment. The KEGG-ZScore-BubblePlot (Figure S1B) identified five pathways directly associated with HBV infection: Arachidonic acid metabolism, Glycerolipid metabolism [8], Neuroactive ligand-receptor interaction, Pancreatic secretion [9], and Pancreatic secretion. After reordering the top 20 significant pathways (Figure S1D), it was found that pancreatic secretion, protein digestion and absorption, and fat digestion and absorption were the most significantly enriched. This suggests that chronic HBV infection may regulate the ‘pancreatic secretion-protein/fat digestion’ cascade pathway and reshape the extracellular microenvironment, thereby providing favorable conditions for continuous viral replication and immune escape. Reactome (Figure 3D) further delineated these processes into trypsin/plasmin-mediated ‘precursor-activity’ cleavage reactions, encompassing the maturation of REG3A/G and HD5 antimicrobial peptides, as well as the activation of the MMPs cascade. This provides new insights into elucidating HBV-host interactions.

### 3.3. Identification of DEmiRNAs

**Figure 4:**
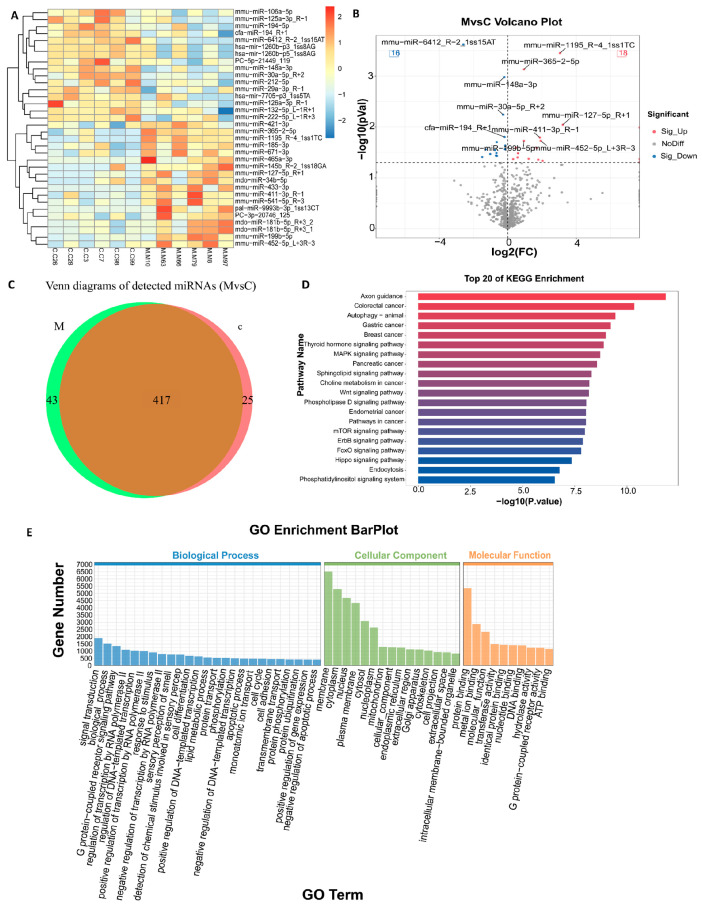
Analysis of *DEmiRNA* expression characteristics (the color bar scale is z-score value: 0 is the mean value, positive values 1 and 2 represent 1,2 standard deviations higher than the mean value, and negative values 1 and 2 represent 1,2 standard deviations lower than the mean value). (**A**) Heat map of *DEmiRNA* clustering; (**B**) Volcano plot of differentially expressed miRNAs; (**C**) Venn diagram of DEmiRNAs; (**D**) Histogram of KEGG enrichment (Top 20) for target genes; (**E**) Histogram of GO enrichment for D*EmiRNA* targets.

The length distribution (Figure S2A) indicated that the primary peaks of *miRNA* in each group were situated between 21 and 23 bp, with comparable expression levels across the groups. The cluster heat map (Figure 4A) showed that the up-regulated and down-regulated *miRNAs* in the comparison group formed independent expression modules, respectively. The Venn diagram (Figure 4C) illustrates that 417 *miRNAs* are co-expressed in the two groups. Among these, 43 and 25 differential miRNAs are unique to the HBV persistent infection mouse group (M group) and the blank control group (C group), respectively. This finding suggests that a substantial number of conservative responses coexist alongside a limited number of group-specific regulatory mechanisms. An in-depth study of the functions of these specific miRNAs will facilitate the discovery of novel therapeutic targets and provide a theoretical foundation for developing new therapeutic strategies against HBV infection. The difference analysis results are as follows (Figure S2B): when the *p* value is less than 0.01, 6 DEmiRNAs (3 ↑, 3 ↓) were detected; when the *p* value was less than 0.05, 44 *DEmiRNAs* (18 ↑, 16 ↓) were detected. When the *p* value was less than 0.1, 71 *DEmiRNAs* (41 ↑, 30 ↓) were detected. *MiR-1195*, *miR-127-5p*, and *miR-6412 R-2*, located at the top of the volcano map, exhibited the three highest enrichment differences and significance levels, with expression changes of approximately +3-fold, +3.5-fold, and −2.5-fold, respectively. These results suggest a strong correlation with HBV infection (Figure 4B). Conserved statistics analysis (Figure S2C) showed the conservation of differential *miRNAs*, which provided a reference for functional annotation and evolutionary research.

We further conducted GO functional annotation on the target genes of the *DEmiRNAs* (Figure 4E). Our analysis revealed that these target genes were significantly enriched in cellular components (CC) and molecular functions (MF), while showing lesser enrichment in biological processes (BP). Additionally, the GO enrichment analysis of the top 20 terms (Figure S2D) indicated that the target genes were notably enriched in MF and CC such as protein binding (MF: GO: 0005515), cytoplasm, nucleus, and membrane. This suggests that these genes may play a role in HBV infection through intracellular protein interactions and the regulation of membrane system structures. KEGG enrichment analysis of the top 20 pathways (Figure 4D) demonstrated that the target genes of *DEmiRNAs* were significantly enriched in HBV-related core pathways. Autophagy in animal models mediates HBV replication and release [10,11]. The MAPK [12,13], Sphingolipid [14], Phospholipase D [15,16], and Phosphatidylinositol [17] signaling pathways are involved in viral envelope formation, lipid microenvironment remodeling, and the entry process. The Wnt [18], mTOR [13], Hippo [19], and Endocytosis [20,21,22] pathways are associated with the expression of the HBx protein. The enrichment analysis of the differential target gene functional pathways indicates that *DEmiRNAs* may play a role in the process of HBV infection by modulating the network associated with ‘lipid environment-signal transduction-endocytosis.’ This finding offers a novel reference for analyzing the molecular regulatory network involved in HBV infection.

### 3.4. Identification of DElncRNAs

**Figure 5:**
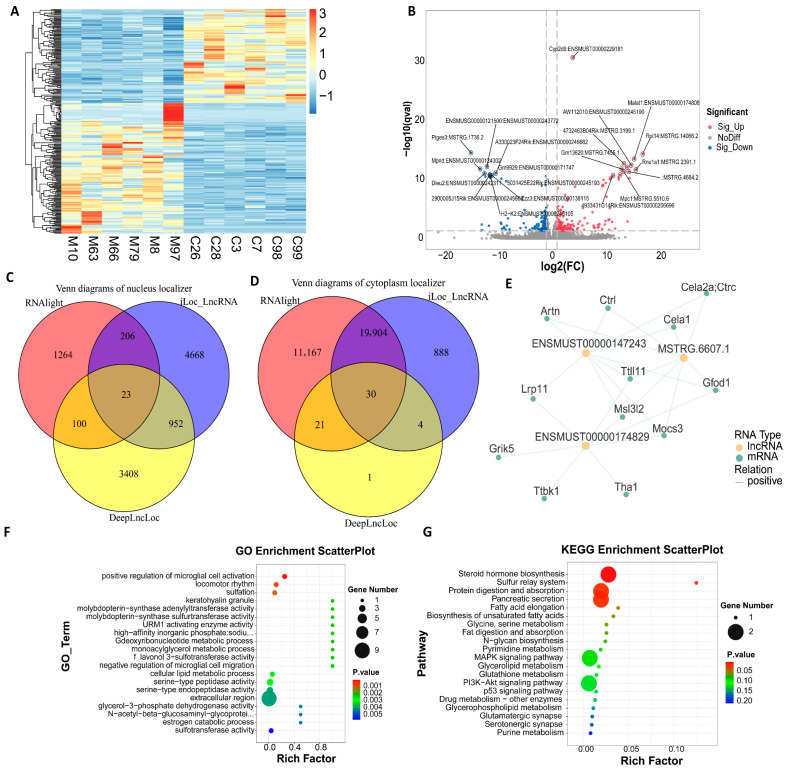
Identification of *DElncRNAs*. (**A**) DElncRNAs clustering heat map (the color bar scale is the z-score value, where 0 is the mean value, positive values 1, 2, and 3 represent 1, 2, and 3 standard deviations higher than the mean value, respectively, and negative values 1 represent 1 standard deviation lower than the mean value); (**B**) gene expression volcano map; (**C**,**D**) subcellular localization of DElncRNAs Venn diagram; (**E**) *DElncRNAs*-protein-coding gene co-expression network diagram; (**F**) GO enrichment bubble diagram of DElncRNAs-protein coding gene TOP20 gene; (**G**) KEGG enrichment histogram of DElncRNAs-protein coding gene TOP20 gene.

Numerous studies have demonstrated that l*ncRNA* can exert precise control over the transcription of adjacent genes through various mechanisms, including cis-regulation, guidance, scaffolding, or baiting, thereby playing a crucial role in disease processes. Consequently, we systematically analyzed the biological functions of *DElncRNAs* in HBV infection to elucidate their potential pathogenic significance. The Circos map (Figure S3A) indicates that the density of lncRNA loci in chromosomes 4, 12, 15, and 17, among others, exceeds the average density across the entire genome. This observation suggests that these regions may harbor a higher abundance of candidate *lncRNAs* associated with HBV infection. Future studies could focus on differential expression screening and functional annotation within these specific regions. The box plot (Figure S3B) showed that the median *lncRNA* prediction score across all samples was below zero, without positive outliers, indicating reliable results suitable for downstream analysis. The overall statistics of DElncRNAs (Figure S3C) revealed the identification of 306 *DElncRNAs*, comprising 184 that were up-regulated and 122 that were down-regulated. The heat map (Figure 5A) clustering results were consistent with the statistical differences, confirming the differences in DElncRNAs expression between groups. The volcano plot (Figure 5B) further emphasizes the genes that are significantly up-regulated and down-regulated. Notably, Malat1 Ptges3 (MSTRG.1736.2) [23] have been identified as being closely associated with HBV infection. Considering the correlation between the subcellular localization of lncRNAs and their mechanisms of action, we employed a combination of LightGBM, Support Vector Machine (SVM), and Convolutional Neural Network (CNN)/Transformer models to predict the localization of DElncRNAs. Our findings reveal that the cytoplasmic subtype of *DElncRNAs* is significantly more prevalent than the nuclear subtype (Figure 5C,D). This suggests that these lncRNAs are inclined to regulate *mRNA* stability and translation through post-transcriptional mechanisms, such as *ceRNAs*, thereby playing a role in HBV infection.

To further explore the regulatory role of *DElncRNAs* in *mRNA* regulation during HBV infection, we extracted three *DElncRNAs* with the highest connectivity (*ENSMUSTO0000147243*, *MSTRG.6607.1*, *ENSMUSTO0000174829*) along with 12 *mRNAs* to construct a sub-network (Figure 5E). Except for *Lrp11* [24], which has been confirmed to be associated with HBV liver disease, the remaining 11 functional genes—including the metabolizing enzyme THA1/GFOD1, proteases CTRL/CELA1/2A, and the microtubule regulatory factor TTLL11—have yet to be studied in relation to HBV. This suggests that novel mechanisms may be concealed within these established genes. The GO enrichment analysis (Figure 5F) indicated that the biological processes were predominantly enriched in the extracellular region. This finding is consistent with previous results and suggests that the key regulatory steps following HBV infection are primarily localized to the extracellular region. The KEGG enrichment analysis (Figure 5G) highlighted significant enrichment of HBV-related signaling pathways, including MAPK [25], PI3K-Akt [26], p53 [27], Glutathione metabolism [28], and Glycerolipid metabolism [28]. This suggests that *DElncRNAs* may regulate host immune responses and metabolic processes through multiple pathways.

### 3.5. Identification of DEcircRNAs

**Figure 6:**
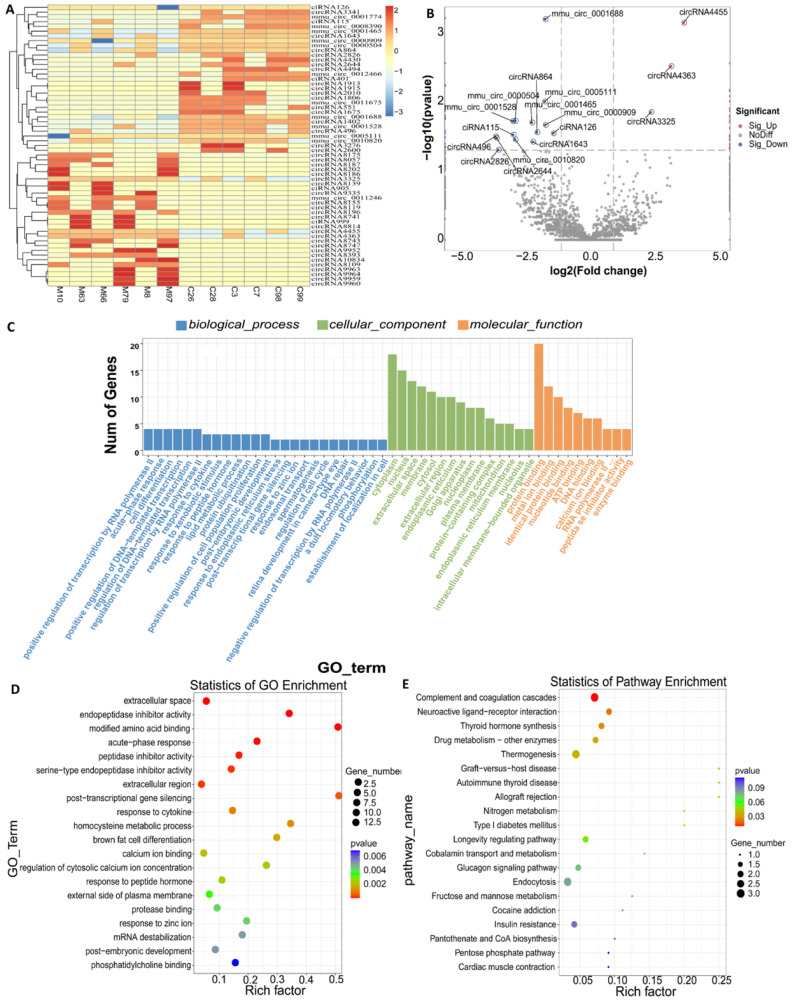
Expression profile analysis of *DEcircRNAs* parental genes (the color bar scale is the z-score value, where 0 is the mean value, positive values 1 and 2 represent 1 and 2 standard deviations higher than the mean value, respectively, and negative values 1, 2, and 3 represent 1, 2, and 3 standard deviation lower than the mean value). (**A**) *DEcircRNA* clustering heat map; (**B**) Differential gene expression volcano map; (**C**) GO enrichment histogram; (**D**) GO enrichment bubble diagram of TOP20 gene; (**E**) KEGG enrichment histogram of TOP20 gene.

The proportion of exons in each sample exceeded 70%, and the combined total of exons and introns was significantly greater than that of intergenic regions (Figure S4A). The FPKM box plot (Figure S4B) indicated that the overall distribution of gene expression across the 12 samples was consistent, demonstrating good repeatability. The chromosomal distribution of the top 20 *circRNAs* (Figure S4C) indicates a widespread distribution throughout the genome. Notably, the number of circRNAs on chromosomes 5, 2, and 1 is significantly enriched, suggesting that these chromosomal regions may exhibit high transcriptional activity or a tendency for cyclization. The results of the aforementioned analysis demonstrate that the quality of the sample data is reliable and meets the criteria for subsequent differential and enrichment analyzes. Hierarchical clustering indicated that the groups were consistently clustered, demonstrating significant separation between them and exhibiting high reproducibility (Figure 6A). A total of 59 *DEcircRNAs* were identified, with 28 exhibiting up-regulation and 31 demonstrating down-regulation (Figure S4D). The volcano plot (Figure 6B) illustrated that *circRNA4455*, *circRNA4363*, and *mmu_circ_0001688* all resided within the significant region (|log2FC| > 2.5 and FDR < 0.05), indicating their potential as focal points for subsequent functional and mechanistic validation.

Subsequently, we performed GO and KEGG enrichment on the differential genes. GO classification revealed that the differentially expressed genes were primarily enriched in CC and MF categories (Figure 6C). Notably, ‘cytoplasm’ and ‘protein binding’ emerged as the most significant terms, indicating that the parental gene products predominantly reside in the cytoplasm and facilitate protein interactions. This finding aligns with the functional characteristics of *circRNAs*, which serve as *miRNA* sponges or *RNA*-protein scaffolds to regulate post-transcriptional networks. Several significant differential genes identified in the GO enrichment analysis (Figure 6D) have been confirmed to be closely associated with HBV infection. Acute-phase and response to zinc ion are associated with acute immune response deficits in chronic HBV infection and persistent viral infection [29,30,31]. Endopeptidase inhibitor activity and serine-type endopeptidase inhibitor activity maintain viral chronic infection mainly by blocking protease activity. Response to cytokine is the key link of host antiviral immunity [31]. extracellular space [32], response to peptide hormone [33], external side of plasma membrane [34], and protease binding are related to the release and replication of viruses. KEGG mapping (Figure 6E) further enriched these genes in HBV immune-metabolic related regulatory pathways.

### 3.6. ceRNA Regulatory Network

**Figure 7:**
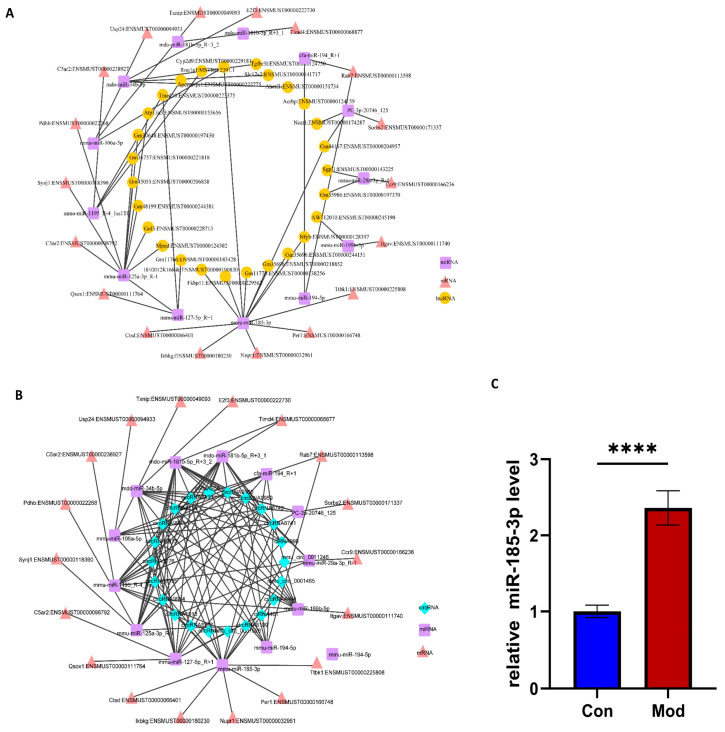
Construction of *ceRNA* network. (**A**) lncRNA-miRNA-mRNA visualization network; (**B**) *circRNA-miRNA-mRNA* visualization network; (**C**) The quantitative PCR (qPCR) assay was conducted to verify the expression levels of *miR-185-3p* in a model of HBV infection (**** *p* < 0.0001).

We integrated significantly enriched differential genes associated with HBV infection-related pathways and predicted *lncRNA/circRNA-miRNA* and *mRNA-miRNA* targeting relationships by intersecting the results from TargetScan (score ≥ 90) and miRanda (Energy < −20). Finally, we screened 18 *mRNAs*, 13 *miRNAs*, 66 *lncRNAs*, and 177 *circRNAs*, constructing and mapping the lncRNA/circRNA-miRNA-mRNA regulatory network. Among the key *miRNAs* screened in this study, *miR-185-3p*, *miR-181b-5p* [32], *miR-194-5p* [35], *miR-29a-3p* [36], and *miR-125a-3p* [37] not only have well-defined human homologs but have also been corroborated by numerous studies as being closely associated with HBV infection and liver pathological processes. Further analysis indicated that *miR-185-3p* exhibited the highest number of associated target genes and *non-coding RNAs* within these two regulatory networks, suggesting its central role in the ceRNA regulatory axis. In the lncRNA-miRNA-mRNA regulatory network, seven *DElncRNAs* including *Cyp2d9*, *Fkbp11*, *Gm11773*, *Gm35696*, *Gm44167*, *Nfyb*, and *1810012K16Rik* were found to be associated with five DEmRNAs (*Ctsd*, *Ikbkg*, *Nupr1*, *Per1*, *Ttbk1*) through *miR-185-3p* (Figure 7A). In the *circRNA-miRNA-mRNA* regulatory network, eleven DEcircRNAs (*circRNA1913*, *circRNA4430*, *circRNA4494*, *circRNA8139*, *circRNA8155*, *circRNA8741*, *circRNA8743*, *circRNA9963*, *circRNA9964*, *mmu_circ_0001465*, and *mmu_circ_0011246*) were significantly associated with the aforementioned five target genes through miR-185-3p. This indicates that these fifteen *DEcircRNAs* may regulate the expression and translation of mRNAs by competitively binding to *miR-185-3p*, thereby modulating the host’s immune response (Figure 7B). We further verified the expression changes in *miR-185-3p* in the HBV infection model through qPCR experiments, which were consistent with the transcriptome analysis (Figure 7C).

## 4. Discussion

The interaction between viral and host factors plays a crucial role throughout the entire life cycle of hepatitis B, significantly influencing the virus’s pathogenicity and the ultimate outcome of the infection. Therefore, it is essential to systematically describe the regulatory network of immune and metabolic reprogramming during HBV infection to elucidate the pathogenesis and develop new antiviral strategies. However, the complex biological characteristics of HBV, the technical challenges associated with constructing cell and animal models, and the intricate multi-level interactions between the virus and host have long restricted related research, leaving the pathogenic mechanisms insufficiently understood. In this study, we established a model of persistent HBV infection in Alb-Cre mice through high-efficiency rcccDNA/Ad transfection. The model mice exhibited continuous positivity for HBsAg and HBeAg in peripheral blood, significantly elevated ALT levels, pathological liver loss, local infiltration of inflammatory cells, and high expression of HBV-DNA. These characteristics confirm that Ad/rcccDNA can promote persistent HBV infection and induce significant inflammatory liver injury in the model mice. This study further integrates whole transcriptome sequencing to systematically analyze the synergistic changes in host immune responses and metabolic reprogramming during HBV infection. It deepens the understanding of the mechanisms underlying HBV-induced immune imbalance and metabolic reprogramming, while also providing a theoretical basis for the development of precise antiviral treatment strategies that are grounded in immune regulation.

In light of the synchronization between changes in host gene expression and HBV infection, we conducted an enrichment analysis on the differentially expressed *mRNAs*. The enriched biological processes can be re-divided and understood according to the ‘host–pathogen’ interaction: Extracellular space, extracellular region, oligosaccharide binding, glucose homeostasis, 2′-5′-oligoadenylate synthetase activity, collagen-containing extracellular matrix, negative regulation of feeding behavior, acute-phase response, digestion, cellular response to corticotropin-releasing hormone stimulus, hormone activity, and omega-hydroxylase P450 pathway are all associated with the host’s subcellular localization, metabolism, immune functions, or endocrine functions; disruption of cell wall in another organism and peptidoglycan binding are the behavioral characteristics of pathogen recognition and destruction of host cell wall; Serine-type endopeptidase activity, serine-type peptidase activity, peptidase activity, proteolysis, oxidoreductase activity, acting on paired donors, monooxygenase activity, and peptidase activity are present in both host immunity and pathogen invasion. Consequently, these functions are classified as shared by both the host and the pathogen. Overall, these gene enrichments indicate that the host simultaneously activates immune responses, metabolism, and endocrine functions in the extracellular space during pathogen invasion. We further focused on the biological processes related to pancreatic secretion, protein digestion and absorption, fat digestion and absorption, influenza A, neuroactive ligand-receptor interactions, starch and sucrose metabolism, arachidonic acid metabolism, maturity-onset diabetes of the young, glycerolipid metabolism, carbohydrate digestion and absorption, steroid hormone biosynthesis, ovarian steroidogenesis, AMPK signaling pathway, longevity-regulating pathways across multiple species, FoxO signaling pathway, glycine, serine, and threonine metabolism, linoleic acid metabolism, retinol metabolism, and insulin resistance, as well as the MAPK signaling pathway. Notably, we found that Arachidonic acid metabolism, AMPK signaling pathway, FoxO signaling pathway, MAPK signaling pathway and Insulin resistance signaling pathway mediated HBV infection and liver inflammation. Liu et al. [38] found that chronic alcohol consumption and HBV infection promote the activation of Tggs through the HBx/SWELL1/arachidonic acid signaling pathway, which exacerbates liver inflammation and induces abnormal lipid metabolism. Song et al. [39] demonstrated that arachidonic acid can inhibit HBV infection in HepG2-NTCP and HepaRG cells. Ye and Niu [40,41] reported that the activation of AMP-activated protein kinase (AMPK) also inhibits lipid synthesis and negatively impacts the metabolic environment of HBV-dependent hepatocytes, thereby limiting viral replication. Furthermore, KEGG enrichment analysis conducted by Li et al. on hepatocyte samples infected with HBV-related hepatocellular carcinoma (HCC) revealed significant upregulation of the FoxO pathway, suggesting its involvement in the development of HBV-related liver disease [42]. Fu et al. [43] further discovered that HBV infection induces hepatocyte death and liver inflammation through the activation of the HBV-STAT3-*miR-328-3p*-FOXO4 axis. It has been established that MAPK activation promotes HBV replication during the early stages of HBV infection [44,45]. Several studies have confirmed that the prevalence of insulin resistance in individuals with chronic HBV infection is significantly higher than that in the general population [46,47]. The specific mechanisms underlying this association warrant further investigation. These findings further substantiate the critical role of significantly differentially expressed genes in HBV in regulating the host immune response.

Although *non-coding RNAs* do not encode proteins, they can regulate *mRNA* expression at pre-transcriptional, post-transcriptional, translational, and protein levels. Therefore, we conducted a further analysis of the regulatory roles of *DEmiRNAs*, *DElncRNAs*, and *DEcircRNAs* in host immune and metabolic reprogramming. The biological processes associated with *DEmiRNAs* are primarily enriched in the membrane, cytoplasm, protein binding, and nucleus. Furthermore, KEGG enrichment analysis identified several signaling pathways that have been confirmed to be closely related to HBV, including autophagy in animals, MAPK, sphingolipid, phosphatidylinositol, Wnt, mTOR, endocytosis, and other related pathways. For example, Xie et al. [48] confirmed that HBV infection induces autophagy, promoting HBV genome replication and persistent infection. Zheng et al. [49] demonstrated that HBV enters hepatocytes via sphingolipid-rich lipid rafts, facilitating receptor aggregation and membrane fusion, and that inhibiting host sphingolipid biosynthesis can significantly reduce HBV replication. Additionally, it has been observed that the levels of phosphoinositides (PI) in the serum of patients with chronic HBV and HepG2 cells are markedly decreased, indicating that the virus enhances its own replication by remodeling host phospholipid metabolism [50]. Li et al. [51] found that mTOR promotes B cell metabolism and immune response through the activation of the HBV particle-TLR2-Akt axis. Furthermore, it has been established that Wnt signaling is closely associated with HBV-related cirrhosis and hepatocellular carcinoma [52,53]. Notably, endocytosis plays a crucial role in HBV infection; viral particles are internalized by hepatocytes through the endocytic pathway, with membrane fusion triggered by the acidification of early endosomes, late endosomes, and lysosomes. This process releases viral nucleic acid into the cytoplasm, thereby initiating the subsequent replication cycle, maintaining persistent infection, and interfering with host immune recognition.

The subcellular localization results of *lncRNA* indicate a stronger involvement in post-transcriptional regulation rather than in epigenetic or transcriptional regulation within the nucleus. To further investigate the regulatory role of *lncRNA* in HBV infection, we selected three *lncRNAs* exhibiting the highest connectivity along with twelve *mRNAs* to construct a co-expression network. However, we identified only one gene (Lrp11) previously associated with HBV liver disease, while the remaining eleven highly connected genes have not been linked to HBV infection. Notably, these genes may represent innovative focal points for our future research. Based on our findings, the biological process of Differentially Expressed *LncRNAs* (DELs) predominantly targeted the extracellular region, as indicated by GO enrichment analysis, which corroborates our previous results. Furthermore, KEGG analysis revealed significant enrichment of HBV-related signaling pathways, including MAPK, PI3K/Akt [54], p53 [55], and glycerolipid metabolism [56]. Notably, both the MAPK and PI3K/Akt signaling pathways were also highlighted in the *mRNA* enrichment results, implying that the regulatory mechanisms associated with these two pathways warrant further investigation during transcription.

In light of the fact that *circRNAs* can regulate transcription, splicing, and translation through *RNA-binding proteins*, and considering that nuclear *EIciRNAs* can synergistically enhance the expression of parental genes in conjunction with U1 snRNP and *RNA* polymerase II, we conducted a systematic analysis of the differential expression of *circRNAs*. The biological processes identified through GO enrichment analysis of *DEcircRNAs* align with those observed in *DElncRNAs* and *DEmiRNAs*, with a consistent emphasis on cytoplasmic functions and protein binding. This suggests that the primary regulatory mechanism of these molecules operates at the post-transcriptional level, indicating a need to focus further on the *ceRNA* regulatory network, rather than solely on intranuclear transcriptional regulation. KEGG enrichment analysis revealed that pathways such as the Complement and Coagulation Cascades, Endocytosis, Pentose Phosphate Pathway (PPP), Insulin Resistance, Glucagon Signaling Pathway, and Drug metabolism—other enzymes are associated with HBV infection. These *DEcircRNAs* may play crucial roles as key genes in HBV infection, host immune regulation, and metabolic reprogramming, thereby facilitating the progression of HBV infection.

To systematically elucidate how differential non-coding *RNA* mediates post-transcriptional regulation of HBV-host immune-metabolic reprogramming through *miRNA*, we screened 18 mRNAs closely associated with HBV infection and constructed ceRNA visualization networks comprising *lncRNA-miRNA-mRNA* and *circRNA-miRNA-mRNA.* Thirteen *miRNAs* functioned as regulatory hubs, while 66 *lncRNAs* and 177 *circRNAs* modulated the availability of these *miRNAs* through competitive binding, thereby controlling the expression of downstream *mRNAs*. The results indicated that these genes are regulated by a diverse array of *non-coding RNAs*. Based on the *ceRNA* regulatory network analysis, this study suggests that *miR-185-3p* plays a central role in the immune regulation associated with HBV. Previous reports indicate that this molecule can significantly inhibit HBsAg expression and viral DNA replication in liver cancer tissues related to HBV [57]. Furthermore, in patients with chronic HBV infection, the expression levels in liver tissue and plasma are significantly correlated with clinicopathological progression [58]. Together, these findings suggest that HBV may evade immune surveillance by modulating the expression of host *miR-185-3p*. The results indicate that *miR-185-3p* not only serves as a marker of the host antiviral response but also has the potential to function as an early warning biomarker for disease progression. Importantly, the downstream target genes of this *miRNA*, including *Ctsd*, *Ikbkg*, *Nupr1*, *Per1*, and *Ttbk1*, have been confirmed to be involved in the key pathological processes of HBV infection. Lucito R et al. [59] confirmed that *IKBKG* serves as a key regulatory subunit of the NF-κB signaling complex, which can be activated by the HBV X protein. This activation promotes the production of inflammatory factors, facilitates viral immune escape, and contributes to liver fibrosis. Several studies have demonstrated that *CTSD* is upregulated in HBV-infected hepatocytes, where it promotes the maturation and secretion of viral particles, and that its expression is further elevated in HBV-related hepatocellular carcinoma tissues, implying roles in both viral replication and liver-cancer progression [60]. Lan et al. [61] demonstrated that the upregulation of *NUPR1* in liver tissues during chronic HBV infection is closely associated with HBV-induced inflammation and may serve as a potential driver of HBV-related liver cancer. Other studies have indicated that the HBV core protein can bind to *PER1*, inhibiting its expression and interfering with the cell cycle and immune response, thereby facilitating the continuous replication of the virus [62]. The activation of TTBK1 enhances the nuclear export of HBV *pregenomic RNA* and promotes viral replication, which is also associated with the cross-activation of inflammatory signals induced by HBV [63].

This study focuses on differential gene screening at the transcriptome level and employs PCR technology to accurately quantify the copy number of key molecules, such as *miR-185-3p*, in liver tissue, thereby evaluating its molecular abundance as a *ceRNA* network hub. However, functional verification of the regulatory network involving miR-185-3p has not yet been conducted. Given the high connectivity of this molecule within the network, future work aims to identify the regulatory axis most closely associated with the pathology of HBV infection. By integrating animal models and cellular experimental systems, and employing functional recovery or deletion strategies (*miR-185-3p* mimics/inhibitors) along with *ceRNA* binding site-specific mutation techniques, the functional interaction mechanisms between *miR-185-3p* and its upstream and downstream molecules will be systematically verified. This approach aims to confirm the pathological correlation and functional significance of the *miR-185-3p* regulatory network within the context of biological functions.

## 5. Conclusions

In this study, we systematically compared the expression differences in *mRNA*, *miRNA*, *lncRNA*, and *circRNA* between mice with persistent HBV infection and normal mice, comprehensively revealing the impact of chronic HBV infection on the host transcriptome. We constructed a regulatory network involving *lncRNA-miRNA-mRNA* and *circRNA-miRNA-mRNA* based on the differentially expressed *mRNAs* closely associated with HBV replication, identifying the key regulatory node, *miR-185-3p*. By interfering with this *miRNA* and its regulated differentially expressed genes, we aim to inhibit HBV replication in the host and mitigate the severe inflammatory response in the liver.

## Figures and Tables

**Table 1:** qPCR primer sequences of HBV-DNA and internal reference gene expression.

Primer Name	Sequence (5′ to 3′)
mus-HBV-F	TATCGCTGGATGTGTCTGCG
mus-HBV-R	GGTGCAATTTCCGTCCGAAG
mus-gapdh-DNA-F	CCCTTCCCACCCTGTTCATC
mus-gapdh-DNA-R	GCTCCTTGCCCTTCCAGATT

**Table 2:** qPCR primer sequences for *miR-185-3p* and internal reference gene expression.

Primer Name	Sequence (5′ to 3′)
*U6-RT-R*	AACGCTTCACGAATTTGCGT
*U6-F*	CTCGCTTCGGCAGCACA
*mus-miR185-3p* *-RT*	GTCGTATCCAGTGCAGGGTCCGAGGTATTCGCACTGGATACGACACCAGA
*mus-miR185-3p* *-F*	CGAGGGGCTGGCTTTCC
*mus-miR185-3p* *-R*	AGTGCAGGGTCCGAGGTATT

## Data Availability

The authors confirm that the data supporting the findings and conclusions of this study are available in the article.

## Supplementary Materials

The following supporting information can be downloaded at: https://www.mdpi.com/article/10.3390/biom15121678/s1, Figure S1: Supplementary Material for *DEmRNA* Identification. (A) The GO enrichment bubble diagram, (B) The KEGG enrichment bubble diagram, (C) The GO enrichment bubble diagram for the top 20 genes, (D) The KEGG enrichment histogram diagram for the top 20 genes; Figure S2: Supplementary Material for *DEmiRNA* Identification. (A) Length distribution histogram of identified miRNAs, (B) Overall statistical histogram of *DEmiRNAs*, (C) Histogram of *miRNA* conservation analysis, (D) Bubble plot of GO enrichment (Top 20) for target genes; Figure S3: Supplementary Material for *DElncRNA* Identification. (A) *lncRNA* type chromosome distribution map, (B) Statistical boxplot for the prediction scores of *lncRNA* in each sample, (C) *DElncRNAs* overall statistical histogram; Figure S4: Supplementary Material for DEcircRNAs Identification. (A) Statistical histogram of *circRNA* type, (B) Box plot of *circRNA* expression distribution, (C) Statistical histogram of chromosome distribution of *circRNA*, (D) Histogram of *circRNA* conservation analysis; Table S1: Quality Control.

## Abbreviations

The following abbreviations are used in this manuscript:

AMPK	AMP-Activated Protein Kinase
BP	Biological Processes
CC	Cellular Components
cccDNA	Covalently Closed Circular DNA
ceRNA	Competing Endogenous RNA
CNN	Convolutional Neural Network
DELs	Differentially Expressed LncRNAs
DEmRNA	Differentially Expressed mRNA
GO	Gene Ontology
HBV	Chronic Hepatitis B virus
HCC	Hepatocellular Carcinoma
KEGG	Kyoto Encyclopedia of Genes and Genomes
MF	Molecular Functions
NAs	Nucleoside (Acid) Analogs
PCA	Principal Component Analysis
PI	Phosphoinositides
PPP	Pentose Phosphate Pathway
qRT-PCR	Quantitative Reverse Transcription Polymerase Chain Reaction
RawData	Raw Sequencing Data
SD	Standard Deviation
SVM	Support Vector Machine
WTS	Whole Transcriptome Sequencing
