# Supplementary material for: Molecular Network Analysis of HBV Persistent Infection from the Perspective of Whole Transcriptome
Source: Biomolecules. 2025 Dec 1;15(12):1678. doi: 10.3390/biom15121678 (PMC12730297; doi:10.3390/biom15121678)
Supplement: Supplementary file 1 [file biomolecules-15-01678-s001.zip › Supplementary Table S1.pdf]

Table S1. Quality Control.

Sample	Raw Data		Valid Data		Valid Ratio (reads)	Q20 %	Q30 %	GC content%
	Read	Base	Read	Base				
C26	66068874	9.91G	59855368	8.98G	90.60	99.99	98.68	52
C28	67820340	10.17G	62158492	9.32G	91.65	99.99	98.67	50
C3	66661892	10.00G	60932570	9.14G	91.41	99.99	98.71	51.50
C7	67715272	10.16G	61332854	9.20G	90.57	99.99	98.66	51
C98	67403866	10.11G	61739338	9.26G	91.60	99.99	98.61	51
C99	67423492	10.11G	60952218	9.14G	90.40	99.99	98.68	52
M10	65720638	9.86G	60180506	9.03G	91.57	99.99	98.74	51.50
M63	57434202	8.62G	52007232	7.80G	90.55	99.99	98.61	52
M66	67090950	10.06G	61671540	9.25G	91.92	99.99	98.71	50.50
M79	60314102	9.05G	55813008	8.37G	92.54	99.99	98.84	50.50
M8	77940530	11.69G	72214842	10.83G	92.65	99.99	98.66	50
M97	68631140	10.29G	58824228	8.82G	85.71	99.98	98.38	63
