# Supplementary figures and images for: Molecular Network Analysis of HBV Persistent Infection from the Perspective of Whole Transcriptome
Source: Biomolecules. 2025 Dec 1;15(12):1678. doi: 10.3390/biom15121678 (PMC12730297; doi:10.3390/biom15121678)

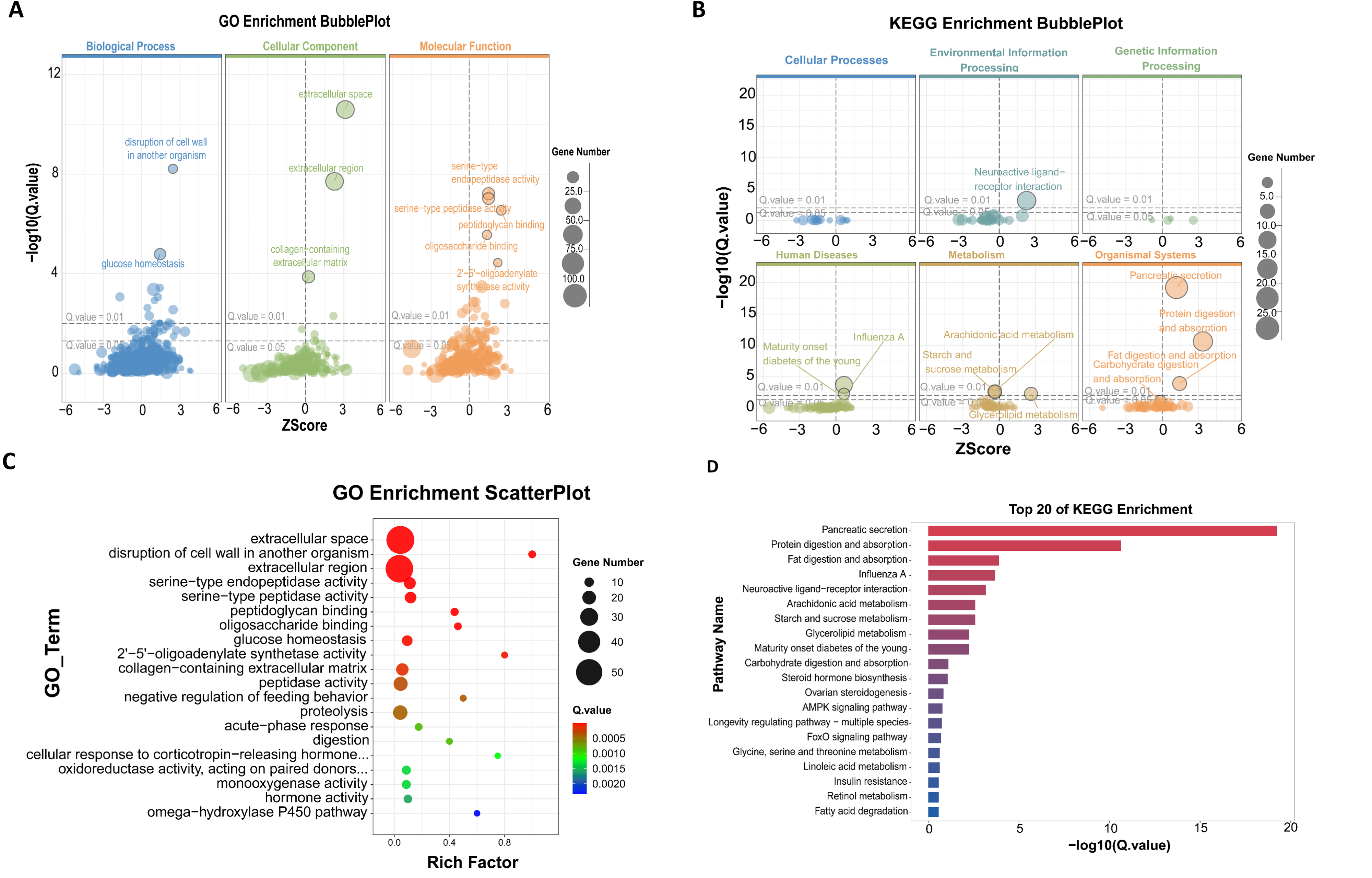

Supplement: Supplementary file 1 [file biomolecules-15-01678-s001.zip › Supplementary Figure S1.tif]

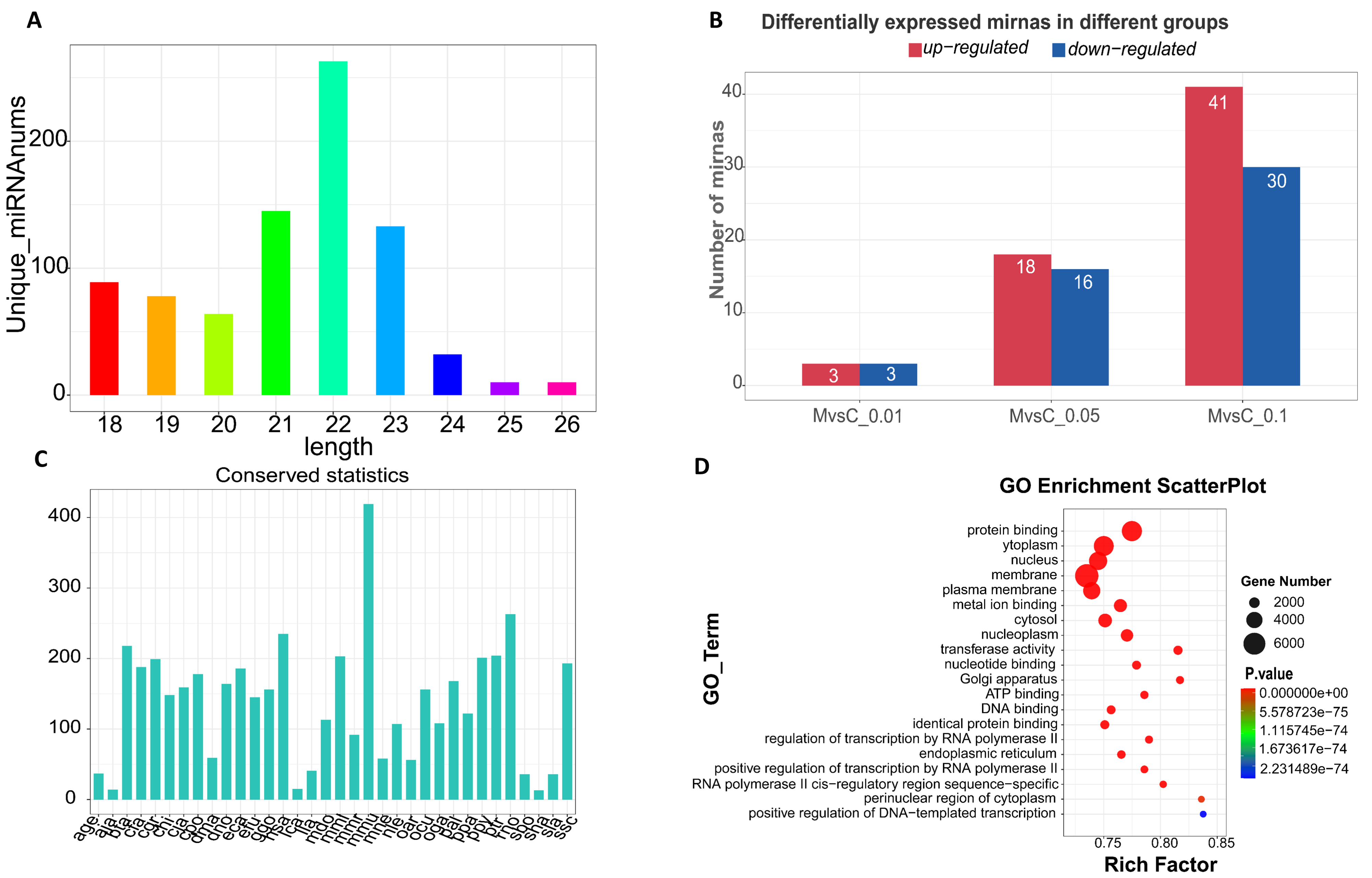

Supplement: Supplementary file 1 [file biomolecules-15-01678-s001.zip › Supplementary Figure S2.tif]

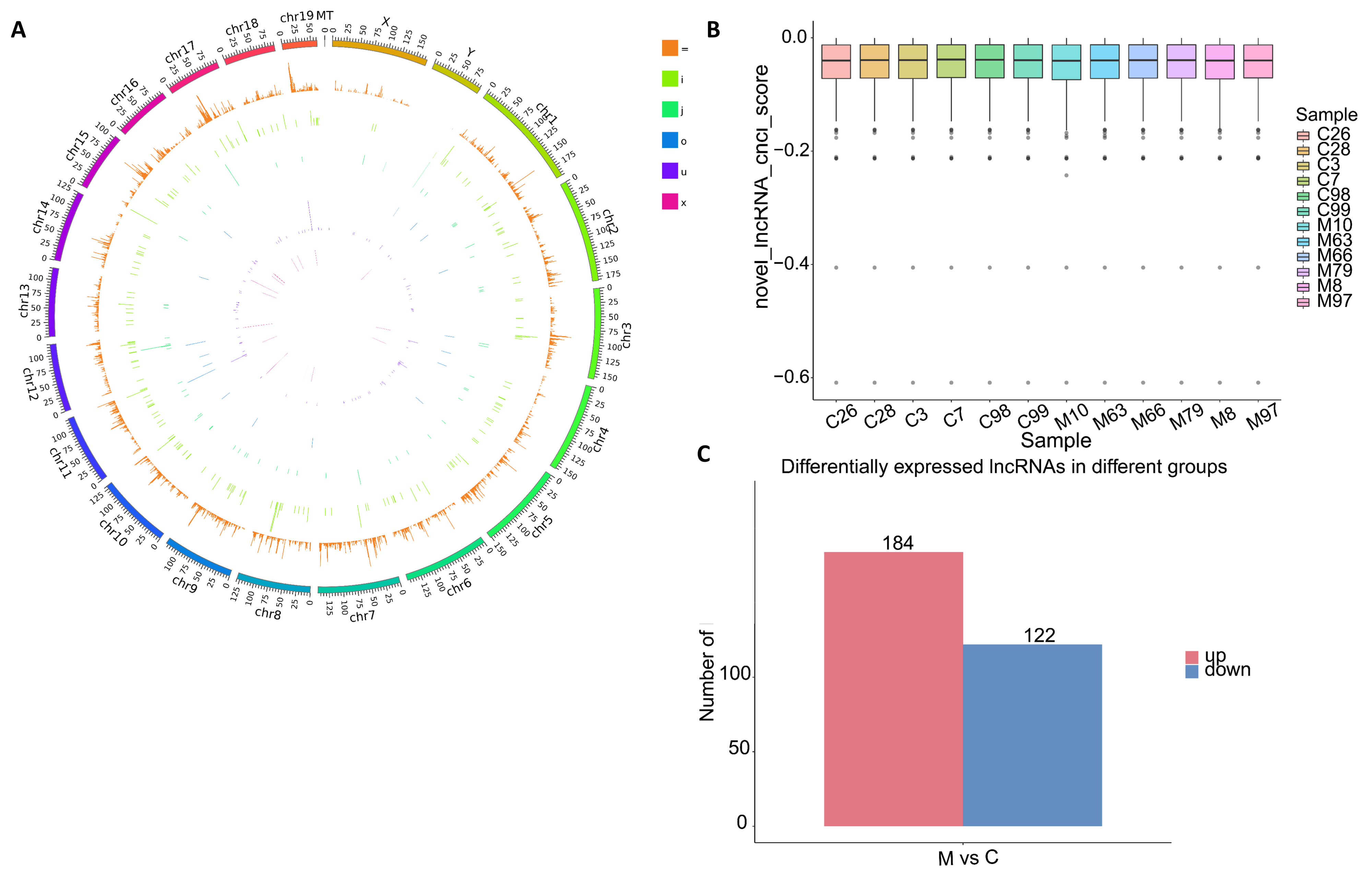

Supplement: Supplementary file 1 [file biomolecules-15-01678-s001.zip › Supplementary Figure S3.tif]

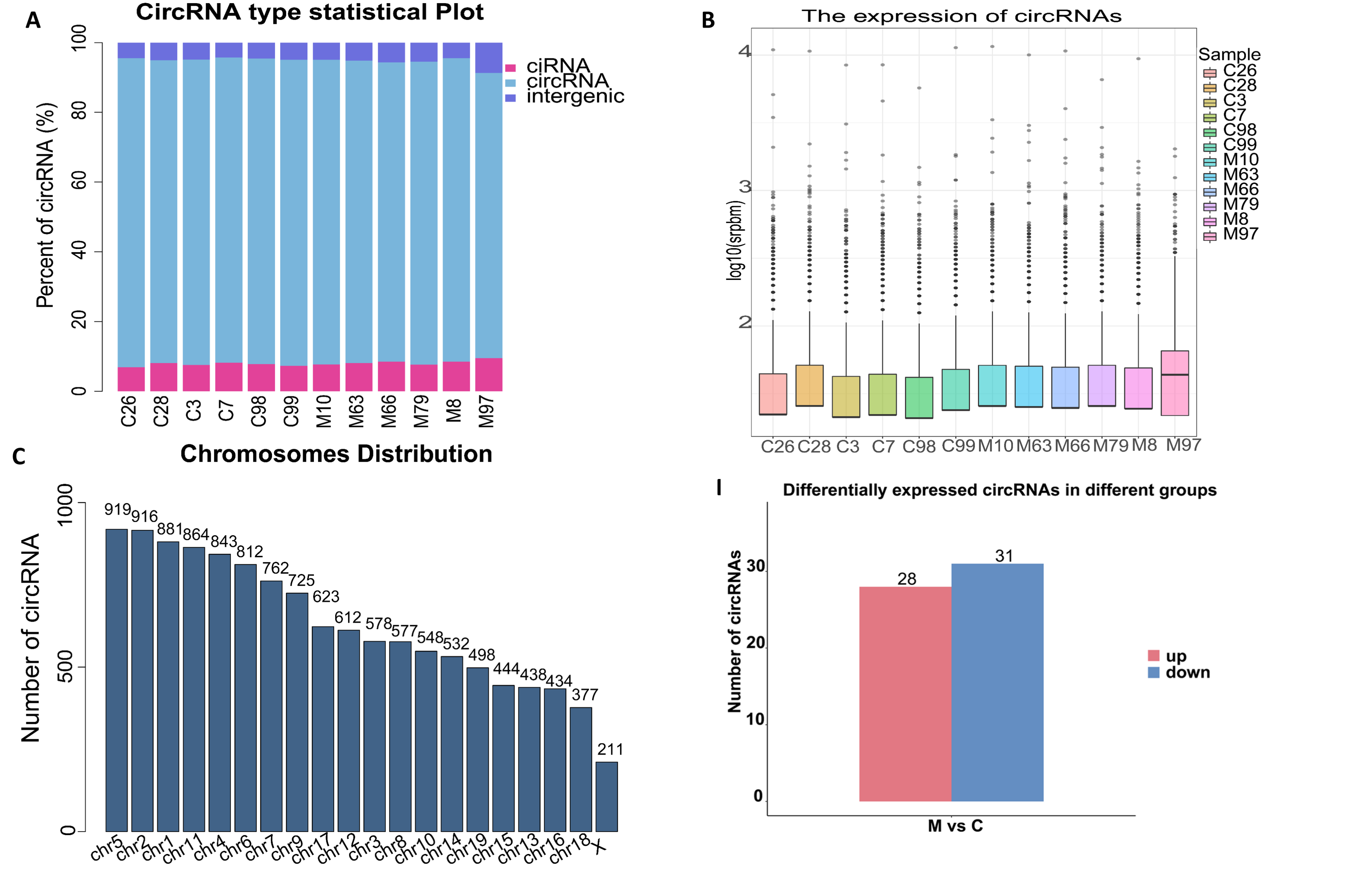

Supplement: Supplementary file 1 [file biomolecules-15-01678-s001.zip › Supplementary Figure S4.tif]
